# Linking prenatal experience to the emerging musical mind

**DOI:** 10.3389/fnsys.2013.00048

**Published:** 2013-09-03

**Authors:** Sangeeta Ullal-Gupta, Christina M. Vanden Bosch der Nederlanden, Parker Tichko, Amir Lahav, Erin E. Hannon

**Affiliations:** Department of Psychology, University of NevadaLas Vegas, NV, USA; Department of Newborn Medicine, Brigham and Women’s Hospital, Harvard Medical SchoolBoston, MA, USA; Department of Pediatrics, Mass General Hospital for ChildrenBoston, MA, USA

**Keywords:** language, music, maternal heartbeat, auditory perception, preterm, development, tempo, isochronous

## Abstract

The musical brain is built over time through experience with a multitude of sounds in the auditory environment. However, learning the melodies, timbres, and rhythms unique to the music and language of one’s culture begins already within the mother’s womb during the third trimester of human development. We review evidence that the intrauterine auditory environment plays a key role in shaping later auditory development and musical preferences. We describe evidence that externally and internally generated sounds influence the developing fetus, and argue that such prenatal auditory experience may set the trajectory for the development of the musical mind.

## Linking prenatal experience to the emerging musical mind

Early experience lays the foundations for the developing musical mind. Hearing emerges prior to birth (Sansavini, 1997) and the acoustic environment *in utero* begins to shape the auditory system much earlier than sensory systems that are not exposed to input until after birth, such as vision. Twenty-five week old fetuses are equipped with structural components of the ear that allow them to hear (Cheour-Luhtanen et al., 1996). Hydrophone recordings reveal that many sounds are measurable in the intrauterine environment, such as the maternal heartbeat, breathing, digestion, and the maternal voice (Dunham, 1990). Even non-maternal speech and song is potentially audible, at or above 60 dB sound pressure level (SPL) and attenuated above 250–500 Hz (Busnel et al., 1992; Gerhardt and Abrams, 1996). Fetal heart rate evidence suggests that during the third trimester fetuses discriminate the speech of their mother from that of a stranger, speech of their native language from a non-native language (Kisilevsky et al., 2003; Kisilevsky and Hains, 2009), and they respond differentially to music and speech (Kisilevsky et al., 2004; Granier-Deferre et al., 2011). Thus, a wide range of maternal and non-maternal sounds are available and potentially audible to the fetus during late pregnancy.

The effects of prenatal auditory experience can be observed among newborns within only a few hours or days after birth. Soon after birth, infants show a strong preference for their mother’s voice over the voice of another female (DeCasper and Fifer, 1980; Cooper and Aslin, 1989; Kisilevsky et al., 2003), their mother’s language over a foreign language (Moon et al., 1993, 2012), and specific passages of speech (DeCasper and Spence, 1986) or music (Hepper, 1991) presented during the final weeks of pregnancy. Thus, even prior to birth human listeners may begin to acquire rudimentary auditory representations that may be considered as the earliest building blocks of the musical mind. Here, we examine the nature of the prenatal auditory input and its effects on auditory preferences, perceptual capacities, and musical skills.

## Fetal experience of externally generated sounds

Prenatal exposure to environmental sounds originating outside the uterus can engender subsequent listening preferences for familiar linguistic and musical stimuli. Newborns prefer to listen to music heard prenatally than to unfamiliar music (Hepper, 1991). Interestingly, this preference diminishes by 21 days after birth, suggesting that prenatal memory for specific sounds may be relatively short-lived. However, other evidence suggests that newborn preferences can generalize to novel sounds that share structural features with sounds heard *in utero*, such as novel speakers uttering familiar passages. Mothers who read a specific nursery rhyme daily during the third trimester had newborns who preferred that nursery rhyme to a novel rhyme; this preference was observed whether the familiar nursery rhyme was read by the infant’s mother or by a female stranger (DeCasper and Spence, 1986). These findings suggest that newborns remember more than the unique characteristics of the mother’s voice, responding to something more general about the familiar passage, such as its prosodic features. The importance of prosody is also implicated by the finding that newborns differentiate between novel, low-pass filtered samples of two languages belonging to contrasting rhythmic classes and they confuse contrasting languages that belong to the same rhythmic class (Nazzi et al., 1998; Ramus et al., 1999). English-learning infants respond similarly to novel utterances of low-pass filtered English and Dutch speech. It is not until 4 or 5 months of age that infants begin to discriminate recordings of their own language from a rhythmically similar foreign language (i.e., English versus Dutch). This evidence suggests that newborns perceive rhythmic features of speech at the earliest stages of development. These early auditory preferences may have important implications for later learning and social development—by 5 months, infants gaze longer at (i.e., show a social preference for) people who speak their native language compared to people who speak a different language or people who speak their native language with a foreign accent (Kinzler et al., 2007).

An important question is whether abnormal experience during this crucial developmental window—for example because of premature birth—influences later language and cognitive development (for review see, McMahon et al., 2012). The later part of gestation is characterized by rapid changes in the neurochemical and structural components of the brain, so infants born prematurely often have diminished brain growth (Guihard-Costa and Larroche, 1990) and medical complications (for review see, Loftin et al., 2010), making it difficult to rigorously compare preterm and full-term infants. However, a longitudinal study found minimal differences in the development of low-level auditory brainstem responses of premature and full-term infants through age six (Jiang, 1995), perhaps because low-level auditory pathways are developed prior to the onset of hearing (Barkat et al., 2011). When perceptual deficits are observed among preterm infants they probably occur at the level of auditory cortex, where abnormal experience with auditory input inappropriate for gestational age would presumably impact the synaptic blooming beginning around 3 months prior to birth (Huttenlocher and Dabholkar, 1997; Fellman et al., 2004; Sanes and Bao, 2009).

Preterm infants show delayed brain responses to features of their native language. For example, greater induced gamma band activity (indicative of selective attention) to the native than to a non-native language was observed among full term 6-month-olds but not among preterm infants until 9 months of age (Pena et al., 2010). This suggests that language differentiation begins 6 months after an infants’ due date rather than 6 months after the onset of exposure to language sounds outside the womb.

Preterm infants also exhibit delayed culture-specific narrowing of phonetic discrimination (i.e., better discrimination of native than non-native speech sounds). While full-term infants exhibit perceptual narrowing by 12 months, preterm infants retain sensitivity to both native and non-native speech contrasts at 12 months of age, as indicated by the presence of an event-related potential component called the mismatched negativity (MMN; Jansson-Verkasalo et al., 2010). Despite no measurable difference in behavioral language outcomes between pre- and full-term infants at 12 months of age, preterm infants show significant deficits in language later in development. For instance, at two years of age, toddlers who were born prematurely lagged behind their peers on measures such as the number of produced words, their development of morphology, and mean length of longest utterances (Jansson-Verkasalo et al., 2010). Further, children’s MMNs at 12 months were significantly correlated with their language abilities at two years, with greater amplitude for non-native speech sounds being associated with greater language deficits. Findings from Pena et al. (2012) suggest preterm infants do not exhibit perceptual narrowing until 15 months of age despite having tested normally for their gestational age on several health (i.e., APGAR scores, cranial size, brain ultrasonography) and auditory (i.e., auditory brainstem response and otoacoustic emissions) measures throughout the course of development, possibly suggesting physical health deficits related to prematurity are unlikely to account for all observed delays. Perceptual learning may begin as soon as hearing is functional, a possibility that is consistent with recent evidence that perceptual narrowing for native-language vowel sounds is already under way prior to birth (Moon et al., 2012). Perceptual narrowing also occurs for musical rhythm and pitch processing (Lynch et al., 1990; Hannon and Trainor, 2007), and there is minimal evidence that additional postnatal experience accelerates culture-specific narrowing for musical pitch processing (Lynch et al., 1995). Auditory experiences during the final trimester may influence the type of input infants attend to based on the preferred or familiar stimuli in the environment (Cooper and Aslin, 1989). These early preferences may bootstrap the acquisition of key features within both the language and the music domains. Although further research is needed, the above findings implicate a maturational process of auditory development that begins *in utero* and is dependent on the acoustic environment and the listening experience, both pre- and postnatally (Werker and Yeung, 2005).

## Fetal experience of internally generated sounds

Internally generated sounds—in particular the continuous, rhythmic sound of the maternal heartbeat—are undoubtedly the most prominent and frequently heard stimuli *in utero*. The prominence of the maternal heartbeat has been implied through preferential sucking paradigms, in which the presentation of a reinforcing sound is contingent on newborns’ increased or decreased sucking rates relative to baseline (Salk, 1962; Spence and DeCasper, 1987; Moon and Fifer, 2000). The effectiveness of the low-pass filtered sound of the maternal heartbeat in reinforcing infant sucking attests to its salience in the intrauterine environment (DeCasper and Sigafoos, 1983). Recordings of the maternal heartbeat more effectively reinforce sucking than do recordings of male speech (Panneton and DeCasper, 1984) or even unfamiliar female speech (DeCasper and Prescott, 2009).

The maternal heartbeat is the fetus’ first metronome. A single beat of the heart is composed of several distinct waves, starting with an initial P wave (in lay terminology, the “lub” of the heart’s rhythmic “lub-dub”) followed by a brief pause, which leads up to the subsequent QRS complex (or the “dub”). Importantly, the P wave and the QRS complex occur in a fixed 1:1 ratio. The distance between any two analogous points on two consecutive heartbeats (for instance, the R-R interval) is also at a constant 1:1 ratio (Benbadis et al., 2007). Because the heartbeat is the first regular and periodic stimulus a fetus hears, it could, in principle, influence subsequent preferences for other periodic auditory stimuli. Nevertheless, no studies have systematically examined whether specific maternal heart rates or beat ratios predict newborn listening *preferences*. Instead, studies examining the role of listening to the maternal heartbeat sounds have focused on the clinical outcomes of such interventions in neonates, typically in addition to presenting the infants with other sounds such as maternal voice and music.

One theory proposes that fetuses become “imprinted” with the isochronous rhythms of the maternal heartbeat leading to a reduction in their overall arousal (Salk, 1960, 1961, 1962). However, evidence of the arousal-modulating effects of the maternal heartbeat is mixed, with some studies revealing no effect on arousal (Tulloch et al., 1964). In an attempt to replicate (Salk, 1960, 1961, 1962), Tulloch and colleagues found no change in weight gain, activity, or formula intake when presented with simulated heartbeat sounds. In this study, the heartbeat sound was audible (as recorded by infants’ startle response at the sound’s offset), but not as loud as the stimuli used in the original study (45 dB versus 85 dB, respectively). Thus, Tulloch et al. postulated that the considerably louder heartbeat sounds used in the Salk studies may have drowned out all background noises, including the sudden noises of airplanes in close proximity to the hospital. On the other hand, if the sounds of the heartbeat, and, specifically, the sounds of the *maternal* heartbeat are important, then not only should heartbeat sounds act to diminish arousal indicating directed attention, but sounds of their own mother’s heartbeat should have greater arousal reduction effects than other heartbeat sounds. Smith and Steinschneider (1975) attempted to test this hypothesis by measuring the maternal heart rate during the last 4 to 6 weeks prior to each infant’s due date. After delivery, newborns were divided into high and low maternal heart rate groups (based on the average heart rate of each infant’s mother). Newborns were coaxed into a state of high arousal (i.e., crying), and were exposed to recordings of high (105 bpm) and low (75 bpm) heart rate samples at 75 dB SPL, as well as silence. Results showed that heart rate sounds—irrespective of whether they were played at tempi consistent with the mothers’ heart rate—resulted in greater proportions of time sleeping and less time crying compared to silence (Smith and Steinschneider, 1975). However, when arousal was measured by the time elapsed until the infant fell asleep, no arousal reduction was found. These studies suggest that benefits of the heartbeat on arousal are perhaps driven by the regular, constant nature of these sounds above the noise floor, providing a salient stimulus that facilitates the filtering out of loud background noises.

A number of studies have shown that multiple maternal sounds, not just the heartbeat, can have soothing effects and short-term clinical benefits for infants, especially in the preterm population. For example, Doheny et al. (2012a,b) reported improved cardiorespiratory stability and reduced episodes of breathing cessation in preterm infants exposed to audio recordings of their mother’s voice and heartbeat, compared to the presentation of regular hospital noise. The combined effects of the mother’s voice and heartbeat are also observed during Kangaroo Care, which encourages parents to hold the newborn close to the chest and maximize skin-to-skin contact, and has been associated with normalizing their body temperature, breathing, heart rate (Ludington-Hoe et al., 2006) and body weight (Charpak et al., 2005). Despite relatively short-term intervention durations, exposure to recordings of the mother’s voice has been associated with increased oxygen saturation levels (Standley and Moore, 1995), improved growth velocity (Zimmerman et al., 2013), fewer episodes of feeding intolerance (Krueger et al., 2010), and reduced hospital stay (Cevasco, 2008). Live interventions such as parental play and song directed toward their preterm infants in the neonatal intensive care unit (NICU) have been particularly potent, yielding reduced heart rate and deeper sleep for 30 minutes after sessions (Arnon et al., 2006), as well as improved breathing and sucking regulation (Loewy et al., 2013). For recorded voices, however, there appears to be no benefit of maternal voice over a stranger’s voice (Segall, 1972), suggesting that voice familiarity is key. Maternal sounds may therefore help optimize pre-term infants’ arousal levels, improve their capacity to maintain a normal breathing pattern, and promote overall health and well-being (Doheny et al., 2012a,b). The extent to which these benefits extend to instrumental music is unclear. Several studies using sung lullabies or simple melodies have shown positive short-term clinical effects for preterm infants (for meta-analysis, see Standley, 2002), however the effects of instrumental music and maternal sounds have not been directly compared, and it is difficult to infer answers from prior work due to the diversity of stimuli (i.e., mother’s or father’s voice, recorded singing combined with uterine sounds, multimodal stimulation) as well as dependent measures (i.e., motor development, weight gain, contingent sucking, or oxygen regulation) (see Chapman, 1978; Malloy, 1979; Standley and Moore, 1995).

## Prenatal experience as a foundation for musical preference

Prenatal exposure to the sounds of language influences infants’ preferences for language, as described above, but this exposure might also generalize to preferences for music. Both vocal and instrumental music have been shown to reflect the characteristic rhythmic patterns of the composer’s native language (Wenk, 1987). Growing evidence suggests that the prosodic features of the native language can influence how adult users of those languages perceive non-linguistic/musical sound patterns such as temporal groups (Iversen et al., 2008), rhythm, or duration (Patel and Daniele, 2003; Hannon, 2009; Marie et al., 2012) and melody (Pfordresher and Brown, 2009; Bidelman et al., 2011). Some of these effects arise during infancy (Yoshida et al., 2010), but others have been observed in newborns. For example, the intonation of newborn cries appears to mimic their native language intonation contours, with French newborns producing rising cries and German newborns producing falling cries (Mampe et al., 2009). Although crying may or may not be a precursor to singing, given the observed parallels between the melodies of song and those of speech (Patel, 2006), it is possible that newborns might also prefer musical melodies that echo the intonation patterns of their native spoken language. Future research on this question is needed.

Although no studies directly examine whether exposure to internal sounds such as the heartbeat can give rise to newborn musical preferences, some evidence is consistent with this possibility. Musical tempo, defined as the number of beats per minute (BPM), is used by composers to manipulate the subjective speed and mood of a piece of music. Tempo can influence adults’ and children’s musical preferences and emotional responses to music, often taking precedence over other musical elements such as instrumentation, vocal vibrato, and genre (LeBlanc, 1982; LeBlanc and McCrary, 1983). Listeners generally prefer a tempo of 100 bpm (Fraisse, 1957, 1967; Drake and Botte, 1993), and this 600 ms interval is often termed the “indifference interval” that is neither “too slow” nor “too fast” (Wundt, 1910; Fraisse, 1963). Listeners have difficulty processing tempi faster than 600 bpm (Friberg and Sundström, 2002; London, 2004) or slower than 24 bpm (Fraisse, 1982; McAuley et al., 2006). Although systematic tempo preferences are not seen in 4-month-old infants (Baruch et al., 2004), infants do show better discrimination of periodic tone sequences that fall in the optimal (i.e., 600 ms) than suboptimal tempo range (Baruch and Drake, 1997).

There is striking individual variation in tempo preferences over the course of the lifespan, with preferred tempo generally decreasing from ~ 200 bpm in young children (Drake et al., 2000; Provasi and Bobin-Bègue, 2003) to 85 bpm in older adults (Vanneste et al., 2001; McAuley et al., 2006). Interestingly, some studies suggest that the natural, spontaneously occurring tempo in adults closely mirrors the adult resting heart rate (Temperley, 1963; Gerstenblith et al., 1977). The decrease in preferred tempo with age is particularly relevant given that the resting heart rate is also known to decrease with age (for review see, Tanaka et al., 2001). Further, when asked to control the tempo of pure tones (Iwanaga, 1995a) or musical pieces (Iwanaga, 1995b), adults pick a preferred tempo closest to their own heartbeat. While this evidence is suggestive of a potential link between heart rate and musical tempo preference, it does not establish causality, and no studies have systematically examined musical tempo preferences among newborns as a function of the maternal or neonatal heartbeat.

Preferences for musical regularity or isochrony (characterized by a 1:1 inter-beat ratio) might also be linked to the very regular, isochronous heartbeat. Adult listeners appear to be strongly drawn towards temporal isochrony in both perception and production. For example, when asked to spontaneously invent or repeat rhythmic patterns, Western adults produce either isochronous intervals (e.g., 1:1) or simple long-short rhythms with a 2:1 ratio (Fraisse, 1982; Essens and Povel, 1985; Povel and Essens, 1985; Essens, 1986; Semjen and Ivry, 2001; Repp et al., 2002; Snyder et al., 2006), a bias that is observed in musicians and non-musicians (Collier and Wright, 1995; Repp et al., 2005). Brain activity also varies depending on whether the participants are producing simple or complex interval ratios, with greater prefrontal activation, suggesting greater reliance on memory resources, observed during production of complex ratios (Sakai et al., 1999).

Preferences and biases towards isochrony are at least partly experience-driven, because isochrony of musical beat and meter is by no means a cultural universal. Music from the Balkans, India, and many other regions contains beat patterns with more complex ratios, such as 3:2 (Merriam, 1981; London, 1995; Clayton, 2000), and listeners from these cultures do not show the same biases towards isochrony (Hannon and Trehub, 2005a; Hannon et al., 2012). Culture-specific biases towards musical isochrony emerge between 6 and 12 months of age and can be altered through exposure to non-isochronous music (Hannon and Trehub, 2005a,b). However, postnatal listening experience cannot account for all aspects of musical temporal processing during infancy. For example, even before 7 months of age, which is prior to culture-specific narrowing of musical rhythm perception, infants exhibit listening preferences and processing advantages for regular over highly irregular sequences (Nakata and Mitani, 2005; Hannon et al., 2011). Despite the difference in listening environments, Turkish and American infants prefer to listen to isochronous (2:1) or moderately non-isochronous (3:2) melodies over highly complex non-isochronous melodies (Soley and Hannon, 2010). Even long-term exposure to non-isochronous meters does not give Turkish adults an advantage over American adults in detecting changes to highly complex meters (Hannon et al., 2012).

The above evidence reveals that humans have musical preferences for patterns that resemble the regularity and tempo of the human heartbeat. It is unclear, however, whether or not these preferences arise because of exposure to the heartbeat *in utero* or due to experience with periodic structures heard after birth. Early auditory experience is crucial for perceiving and producing rhythmic structures, as evidenced by individuals with congenital beat deafness (Phillips-Silver et al., 2011). Pre- and post-natally, infants are exposed to periodic biological phenomena, such as breathing and locomotion. One possibility is that periodic movement such as locomotion may be the primary driver of tempo preferences, a proposal that is consistent with robust connections between auditory and motor processes in rhythmic behavior, and the finding that preferred tempi decrease with age and body size (Drake et al., 2000; Trainor, 2007). However, tempo biases and beat induction are evident during infancy even prior to locomotion (Baruch and Drake, 1997; Winkler et al., 2009), suggesting that maternal movement may be more influential than the infant’s own movements, at least during infancy (Phillips-Silver and Trainor, 2005, 2007). To provide more compelling evidence of a link between prenatal exposure and musical preferences, it would be necessary to measure maternal heart rate or walking speed during the last trimester and compare it with infants’ preferred tempo and regularity at birth. Moreover, it would be ideal to directly manipulate the prenatal auditory environment through exercise manipulations, instructions to the mother, and so forth as prior studies have done for language stimuli (DeCasper and Spence, 1986).

The musical brain is shaped by its auditory environment during development. Auditory experience does not commence at birth, but during the months prior to birth. Thus, the unique intrauterine auditory environment, dominated by the isochronous maternal heartbeat as well as filtered but structured linguistic and musical input from the external environment, must be considered for its role in influencing perception of and preferences for musical patterns during infancy that feed into later development. Given the large corpus of evidence that prenatal auditory experience plays a critical role in language development, further research should investigate its role in the development of musical preferences. Moreover, the importance of auditory experience during the last trimester of pregnancy has critical implications for designing neonatal intensive care units for preterm infants, who are deprived of a typical prenatal auditory environment.

## Conflict of interest statement

The authors declare that the research was conducted in the absence of any commercial or financial relationships that could be construed as a potential conflict of interest.
